# The oculocerebrorenal syndrome of Lowe: an update

**DOI:** 10.1007/s00467-016-3343-3

**Published:** 2016-03-24

**Authors:** Arend Bökenkamp, Michael Ludwig

**Affiliations:** 1 Department of Pediatric Nephrology, VU University Medical Center, de Boelelaan 1112, 1081 HV Amsterdam, The Netherlands; 2 Department of Clinical Chemistry and Clinical Pharmacology, University of Bonn, Bonn, Germany

**Keywords:** Congenital cataract, Cognitive and behavioral impairment, *OCRL* gene, Oculocerebrorenal syndrome, Inositol-polyphosphate 5-phosphatase, Proximal tubulopathy, Renal Fanconi syndrome

## Abstract

The oculocerebrorenal syndrome of Lowe is a rare X-linked multisystemic disorder characterized by the triad of congenital cataracts, intellectual disability, and proximal renal tubular dysfunction. Whereas the ocular manifestations and severe muscular hypotonia are the typical first diagnostic clues apparent at birth, the manifestations of incomplete renal Fanconi syndrome are often recognized only later in life. Other characteristic features are progressive severe growth retardation and behavioral problems, with tantrums. Many patients develop a debilitating arthropathy. Treatment is symptomatic, and the life span rarely exceeds 40 years. The causative oculocerebrorenal syndrome of Lowe gene (*OCRL*) encodes the inositol polyphosphate 5-phosphatase OCRL-1. *OCRL* variants have not only been found in classic Lowe syndrome, but also in patients with a predominantly renal phenotype classified as Dent disease type 2 (Dent-2). Recent data indicate that there is a phenotypic continuum between Dent-2 disease and Lowe syndrome, suggesting that there are individual differences in the ability to compensate for the loss of enzyme function. Extensive research has demonstrated that OCRL-1 is involved in multiple intracellular processes involving endocytic trafficking and actin skeleton dynamics. This explains the multi-organ manifestations of the disease. Still, the mechanisms underlying the wide phenotypic spectrum are poorly understood, and we are far from a causative therapy. In this review, we provide an update on clinical and molecular genetic findings in Lowe syndrome and the cellular and physiological functions of OCRL-1.

## Introduction

The classic form of the oculocerebrorenal syndrome of Lowe (OMIM #309000), first described by Lowe et al. in 1952 [1], is characterized by the triad of congenital cataracts, severe intellectual impairment, and renal tubular dysfunction with slowly progressive renal failure [2, 3]. Other features include postnatal growth retardation independent of kidney function, areflexia, nontender joint swelling, subcutaneous nodules, and arthropathy, which can be observed in about 50 % of adult patients [3].

Lowe syndrome is caused by variants in the *OCRL* gene on chromosome Xq25-26, which encodes OCRL-1, an inositol polyphosphate 5-phosphatase [4]. Interestingly, variants in *OCRL* have also been found in some patients with a Dent-like disease (OMIM #300009), now called Dent disease type 2 (Dent-2; OMIM #300555), raising the question of how variants in the same gene could cause two seemingly distinct diseases [5, 6]. In fact, many Dent-2 patients show mild extra-renal features of Lowe syndrome, suggesting that Dent-2 disease represents a mild form of Lowe syndrome [7, 8].

Here, we review the phenotypic features of Lowe syndrome, the molecular genetics of *OCRL* variants, and the current understanding of the physiological functions of OCRL-1.

## Prevalence

Based on the observations of the American Lowe’s syndrome Association (LSA) and the Italian Association of Lowe’s Syndrome (AISLO), the prevalence of Lowe syndrome has been estimated to be 1 in 500,000 in the general population [3].

## Clinical manifestations and management

Lowe syndrome is a multisystem disorder involving mainly the eyes, the central nervous system (CNS), and the kidneys. The manifestation of different symptoms over time is summarized in Table 1.

**Table 1:** Typical age at manifestation of symptoms or complications of Lowe syndrome

Age at onset	Manifestation
Prenatal	Cataract
	Elevated alpha-fetoprotein
Neonatal	Cataract
	Muscle hypotonia
	Absent deep tendon reflexes
	Elevated creatinine kinase/lactate dehydrogenase
	Low-molecular-weight proteinuria
1–3 months	Fanconi syndrome
Infancy	Glaucoma
	Growth retardation
	Developmental delay
Childhood	Behavioral abnormalities
	Corneal scarring, keloids
	Tubulointerstitial fibrosis/glomerulosclerosis
Adolescence	Scoliosis
Adulthood	Arthropathy
	End-stage renal disease
No specific age	Convulsions
	Platelet dysfunction

### Eyes

Dense congenital bilateral cataract is a hallmark of Lowe syndrome and present at birth [3, 9, 10]. Cataracts develop early in embryogenesis due to defective formation and subsequent degeneration of the primary posterior lens fibers [9] and have even been demonstrated on prenatal ultrasound images [11]. Severe glaucoma with buphthalmos requiring surgical management is observed in around 50 % of Lowe syndrome patients, usually in the first year of life but possibly as late as in the second or third decade [10, 12]. Corneal scarring and keloids develop without prior trauma in about 25 % of patients usually after the age of 5 years [13, 14]. Corrected visual acuity is rarely better than 20/100 [15], partly due to a primary retinal dysfunction [13]. Management includes early lens extraction and prescription of eyeglasses, while surgical lens implants are not recommended [3]. Lewis et al. also advise that contact lenses not be prescribed because of the risk of corneal keloid formation []. Ocular tone should be tested regularly and glaucoma treated as necessary.

Detailed ophthalmological examination of patients with Dent-2 disease may reveal discrete peripheral opacities between the nucleus and cortex that are clinically asymptomatic [7]. Several recent reports have described the absence [17, 18] or late manifestation [19, 20] of cataract in patients with *OCRL* variants, thereby underlining the phenotypic continuum between Dent-2 disease and Lowe syndrome.

### Nervous system

Both the central and the peripheral nervous system are involved in Lowe syndrome, and it is their involvement which causes the greatest disease burden of the illness [13]. Although less well documented, Dent-2 patients with the mild phenotype of *ORCL* variants may also show some developmental delay [5, 7].

#### Muscle hypotonia

The first clinical symptom is severe neonatal hypotonia, often in the absence of deep tendon reflexes [3, 21]. The hypotonia is of central origin, although muscle biopsy in two brothers demonstrated selective type-1 fiber atrophy resembling congenital fiber type disproportion myopathy [22], and creatine kinase and/or lactate dehydrogenase levels are typically elevated in Lowe syndrome and to a lesser extent in Dent-2 disease [7, 23, 24]. Decreased motor tone results in delayed motor milestones (75 % of patients achieve independent ambulation by the age of 6–13 years) [15].

#### Intellectual disability

The majority of Lowe syndrome patients have severe intellectual impairment with a mean IQ in the range of 40–54. Still, in a study on 47 patients with Lowe syndrome, 25 % had an IQ of >70 [25].

#### Seizures

Seizures occur in up to 50 % of Lowe syndrome patients [13, 26]. There is no specific seizure type.

#### Behavioral abnormalities

Patients with Lowe syndrome have a characteristic pattern of behavioral abnormalities. More than 80 % of patients show stubbornness, aggression, irritability, temper tantrums, and complex repetitive purposeless movements (e.g., hand flapping) that interfere with adaptive functioning and are significantly worse than those observed in other visually impaired or comparably mentally retarded individuals [27]. There is a high prevalence of self-injury associated with repetitive and impulsive behavior [28]. Some evidence suggests that the most difficult period for behavior problems is between the ages of 8 and 13 years. Drugs, such as neuroleptics, antidepressants, stimulants, and benzodiazepines, are only partially effective. More promising results have been reported with clomipramine, paroxetine, and risperidone [3, 15].

#### Neuroradiological and neuropathological features

Cranial magnetic resonance (MR) imaging may demonstrate mild ventriculomegaly and hyperintense lesions on T2-weighted images that are distributed in the periventricular and deep white matter. These lesions correspond to perivascular lacunes and are undetectable until cerebral myelination is well advanced [29]. They appear to be stable in size and location and have no clinical significance [13].

Onur et al. [30] reported a tigroid pattern with hypointense radially oriented stripes within the hyperintense cerebral white matter on T2-weighted images. This pattern of demyelination has also been described in Pelizaeus–Merzbacher disease, globoid cell leukodystrophy, and metachromatic leukodystrophy. Proton MR spectroscopy in Lowe syndrome has shown prominent myoinositol peaks suggesting the presence of gliosis [31].

Neuropathological findings are variable and non-specific and may include ventriculomegaly, brain atrophy, cerebellar hypoplasia, pachygyria, polymicrogyria, aberrant neuronal migration, subependymal cysts, and cysts located in the white matter [13].

### Kidney

The renal phenotype of Lowe syndrome is characterized by proximal tubular dysfunction [2, 7] and slowly progressive renal failure which often leads to end-stage renal disease (ESRD) in the second or third decade. Unlike congenital cataract, the renal tubular dysfunction is not always present at birth; rather, it usually manifests within the first weeks to months [23, 32].

#### Low-molecular-weight proteinuria

**Fig. 1:**
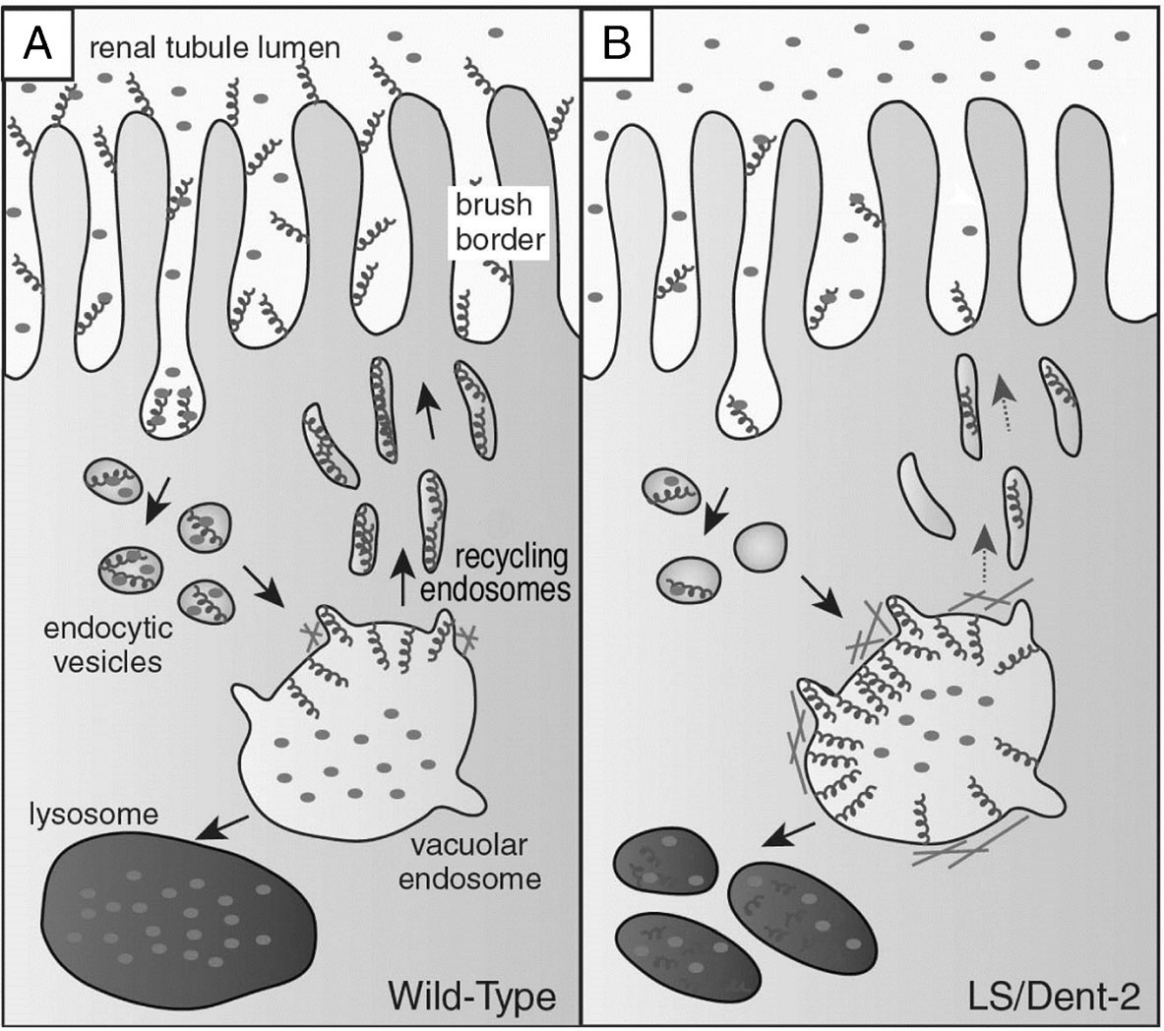
Pathogenesis of low-molecular-weight (LMW) proteinuria in Lowe syndrome. LMW proteins (*filled circles*) are internalized after binding to megalin (*helices*) on the brush border of proximal tubular cells. **a** In the wild-type, the megalin–LMW protein complex in the endosome dissociates at low pH, followed by the recycling of megalin to the cell surface and delivery of the LMW proteins to lysosomes for degradation. **b** In Lowe syndrome/Dent-2 disease, megalin trafficking to the cell surface is impaired. Due to the aberrant accumulation of actin at the endosomal membrane, megalin is retained in the endosome and mis-sorted to the lysosome instead of being recycled to the brush border via recycling tubules (modified from Mehta et al. [33] with permission)

Low-molecular-weight (LMW) proteinuria is a cardinal finding in Lowe syndrome and is observed in all patients. This condition reflects impaired megalin–cubulin receptor-mediated endocytosis in the proximal tubule [33] (Fig. 1), and it has been detected directly after birth prior to any other symptom of proximal tubular dysfunction [34]. Retinol binding protein in particular is a highly sensitive marker for the impairment of tubular protein absorption, presenting in this context with a mean elevation of approximately 1000-fold above the upper limit of normal [2, 35]. Alternative markers are alpha-1 and beta-2 microglobulin, the latter being unstable at a urine pH of <5.5. Although LMW proteins are the major constituents of proteinuria in Lowe syndrome patients, urinary albumin excretion is also elevated. This reflects defective re-absorption via the megalin receptor pathway of some of the 3.3 g of albumin passing through the intact glomerular barrier per day [36]. Total proteinuria is in the nephrotic range (>1 g/m^2^/day) [37] in more than one-half of the patients, but serum albumin concentrations are normal [23]; this also applies to patients with the milder phenotype of Dent-2 disease [38].

#### Aminoaciduria

Generalized aminoaciduria is observed in around 80 % of patients with classic Lowe syndrome, but only in about one-half of patients with Dent-2 disease [7]. There is considerable inter-individual variation in amino acid excretion [2]. Charnas et al. noted that branched-chain amino acids were spared [23].

#### Lysosomal enzymuria/hyperenzymemia

Although* N*-acetyl-ß-d-glucosamine levels have been reported only infrequently, all patients with Lowe syndrome who have been tested showed increased levels of this enzyme [2, 34]. Nielsen et al. [39] demonstrated that filtered lysosomal enzymes are physiologically reabsorbed in the proximal tubule via megalin, suggesting that impaired uptake is a common mechanism of LWM proteinuria and lysosomal enzymuria in Lowe and other forms of the Fanconi syndrome. Ungewickell and Majerus reported a 1.6- to 2.0-fold increase in plasma levels of seven lysosomal enzymes in Lowe syndrome patients [40], which is attributed to disturbed lysosomal enzyme trafficking [41]. Mistargetting of lysosomal enzymes might cause shedding of lysosomal enzymes via the apical membrane of proximal tubular cells and might also explain tissue damage in Lowe syndrome patients.

#### Hypercalciuria/nephrocalcinosis

Hypercalciuria is a common finding in patients with Lowe syndrome and in Dent-2 patients [2, 7, 42, 43] and is observed in approximately 80 % of patients. Urinary calcium excretion is about twofold higher than the age-appropriate upper limit of normal and is independent of age [2].

The pathophysiology of hypercalciuria in patients with *OCRL* defects has not been fully elucidated. It is tempting to extrapolate findings from patients with Dent disease type 1 (Dent-1 disease) who harbor defects in *CLCN5* leading to impaired megalin–cubulin receptor-mediated endocytosis in the proximal tubule. In a *Clcn-5* knock-out model, Günther et al. demonstrated loss of vitamin D-binding protein and increased distal delivery of parathyroid hormone (PTH) [44]. While the former reduces 25-hydroxyvitamin D3 levels, the latter stimulates 1-alpha hydroxylase, leading to increased formation of 1,25-dihydroxyvitamin D3 and resulting in hyperabsorptive hypercalciuria. Another line of evidence indicates a direct effect of OCRL-1 on intestinal calcium transport [45] via the intestinal calcium channel TRPV6 (transient receptor potential, vanilloid subfamily, subtype 6), which is regulated by OCRL-1 and mediates 1,25-dihydroxyvitamin D3 action in intestinal epithelial cells.

Nephrocalcinosis/nephrolithiasis is present in approximately one-half of Lowe syndrome patients [2, 7]. Stones are composed of calcium oxalate and calcium phosphate [2]. Similar to patients with *CLCN5* variants [46], the presence of nephrocalcinosis/nephrolithiasis has not been found to be related to calciuria or to age [2].

There are no data on the treatment of nephrocalcinosis in patients with Lowe syndrome. Thiazide diuretics have been used to decrease calcium excretion in Dent-1 patients [47], but the use of diuretics in the setting of renal potassium loss has to be weighed against the risk of hypokalemia and hypovolemia. Potassium citrate may be useful as it corrects both hypokalemia and metabolic acidosis and has been shown to retard nephrocalcinosis in an animal model of Dent-1 disease [48].

#### Acidosis

Hyperchloremic metabolic acidosis is a common finding in Lowe syndrome and is observed in 33–82 % of patients [2, 7, 32, 49]. Even in non-acidotic patients plasma total carbon dioxide concentration is typically found at the lower end of normal [2]. Renal tubular acidosis (RTA) appears to be less prevalent in patients with Dent-2 disease [7]. Although most reports classify renal tubular acidosis as type 2 (i.e., proximal RTA), Lowe et al. reported that decreased ammonia production differentiated patients with Lowe syndrome from those with other forms of the renal Fanconi syndrome in whom a strongly increased amount of ammonia was detected [1].

#### Phosphaturia

As with other proximal tubular functions, data on the prevalence of phosphate wasting vary considerably in the literature. Böckenhauer et al. [2] reported phosphate wasting in three of 15 patients with Lowe syndrome, with two of the former requiring supplementation. In other studies, however, phosphate wasting has been reported to be present in approximately 40–50 % of Lowe syndrome patients [7, 32, 43, 49]. Abbassi et al. [32] reported that hypophosphatemic rickets was observed in 50 % of their untreated patients with Lowe syndrome, usually manifesting at the age of 1 year. As discussed by Böckenhauer et al. [2], any assessment of phosphaturia may be complicated by the often-present elevated PTH levels. Tubular maximum for phosphate reabsorption/glomerular filtration rate (TmP/GFR) values in their series were obtained while PTH levels were normal, and seven of the 16 patients investigated required 1-OH cholecalciferol substitution to keep the level of PTH in the normal range.

#### Glycosuria

The most striking difference with other forms of the renal Fanconi syndrome is the absence of glycosuria in the vast majority of patients with Lowe syndrome [2, 7, 32, 43, 49].

#### Poor renal accumulation of ^99m^technetium-dimercaptosuccinic acid

99^m^-Technetium-dimercaptosuccinic acid (^99m^Tc-DMSA) scans are used to assess tubulointerstitial integrity and to detect focal scarring. DMSA passes through the glomerular filtration barrier and enters the proximal tubular cells via the megalin–cubulin system [50]. Consequently, defective accumulation of ^99m^Tc-DMSA is a common finding in patients with proximal tubular damage and has been reported in patients with Lowe syndrome [50, 51], with Dent-1/Dent-2 disease [52, 53], as well as with other forms of the renal Fanconi syndrome [54].

#### Progressive renal failure

Slowly progressive renal failure is a hallmark of Lowe syndrome and leads to ESRD in adulthood. Monitoring of kidney function using serum creatinine-based estimates of GFR may result in an overestimation of the GFR due to the decreased muscle mass of patients with Lowe syndrome. Böckenhauer et al. [2] recalibrated the Schwartz-equation using ^51^Cr-EDTA clearance and derived a* k* value of 26 (as compared to 36 in the most recent version of the Schwartz-equation [55]). In view of the abnormal muscle mass of patients with Lowe syndrome, cystatin C-based estimates of GFR [56] should be the method of choice in this patient group. Due to calibration issues, only recent GFR estimating equations based on the reference material provided by the International Federation for Clinical Chemists [57, 58] should be used.

Cross-sectional [7, 49] as well as serial [2] studies have demonstrated a slowly progressive decline in GFR starting from low-normal values [chronic kidney disease (CKD) stage 1–2] in the first year of life to CKD stage 4–5 in the second to fourth decade of life. There is wide intra- and inter-individual variation in decline in GFR. Intra-individual variability in GFR may reflect changes in hydration from salt loss and decreased concentrating capacity, as observed in 21 of the 23 patients in the series of Charnas et al. [49]. Tricot et al. reported a patient who developed ESRD at the age of 49 years [59] and was started on chronic ambulatory peritoneal dialysis. Although transplantation was considered, it is not clear from the report whether this patient actually did receive a renal transplant.

Patients with the Dent-2 disease phenotype have better preserved kidney function than those with Lowe syndrome. In a pediatric series of 25 children with Dent-2 disease, eight had CKD stage 2. Unlike in Lowe syndrome, there was no correlation between preserved kidney function and age [7]. There are very limited data on adults [60], and to the best of our knowledge there are no reports of a patient with Dent-2 disease and ESRD.

The pathogenesis of progressive renal failure in Lowe syndrome is not entirely clear. Renal biopsy shows a characteristic course, starting with normal biopsy findings in children aged 1 or 2 years [61, 62], followed by tubular dilation with proteinaceous casts at age 3–5 years [32] and increased glomerular cellularity and focal glomerular sclerosis as well as diffuse tubulointerstitial fibrosis in older children [32, 59, 61, 62]. This progression is in line with histological findings in patients with Dent-1 disease due to variants in *CLCN5* [63, 64]. Renal biopsy in four patients with Dent-2 disease (age at biopsy 4–16 years) was unremarkable in three patients [38, 52, 60] and showed isolated focal-segmental glomerulosclerosis in one patient [65].

The fact that renal tubular dysfunction precedes the decline in renal function suggests that glomerulosclerosis results from progressive renal tubular injury, leading to tubulointerstitial fibrosis [59, ]. Of note, Norden et al. demonstrated tubular wasting of a wide variety of polypeptides, hormones (e.g., insulin, growth hormone, insulin-like growth factor 1), and chemokines (e.g., monocyte chemoattractant protein 1) in patients with defective absorption of LMW protein [67]. Some of these molecules have been implicated in the pathogenesis of tubulointerstitial fibrosis and might play a role in patients with Dent disease and Lowe syndrome.

### Other manifestations

#### Musculoskeletal

Musculoskeletal complications can arise from the principal manifestations of Lowe syndrome, i.e., hypotonia and renal disease, or as a unique manifestation of the underlying disorder [13]. Hypotonia contributes to joint hypermobility, and decreased movement fosters the development of contractures and osteopenia. About one-half of the patients develop scoliosis [15], which often progresses post-puberty [13].

Osteopenia is almost universally present in patients with Lowe syndrome and may be worsened by untreated acidosis and renal phosphate wasting [49]. Treatment with 1-OH vitamin D is often required to normalize increased PTH levels [2]. However, even in the presence of well-corrected Fanconi syndrome, some patients have repeat pathologic bone fractures with poor healing []. There is one report describing the combination of intravenous pamidronate treatment with growth hormone and the standard therapy of renal Fanconi syndrome in a pre-pubertal 17-year-old Lowe syndrome patient with multiple fractures, extreme stunting, and osteopenia [68]. Bone mineral density increased from −7.3 to −3.3 standard deviation over a period of 3 years. During this treatment the patient did not develop new fractures.

Tenosynovitis, arthritis, and a debilitating arthropathy are frequent complications of Lowe syndrome [69, 70] and have been reported in one-half of the patients over 20 years of age [15]. Clinical manifestations are palmar and plantar fibrosis, focal nodules, non-tender swelling of multiple interphalangeal and metacarpal joints, ankles, and wrists leading to flexion contractures and, eventually, bone erosions. Synovial biopsy shows rubbery tissue without an inflammatory infiltrate and fibrous tissue containing fibrillary material [70]. Zhu et al. have recently demonstrated abundant expression of *OCRL* in normal cartilage, which was downregulated in a mouse model of osteoarthritis and could be restored by intraarticular injection of *ORCL*-encoding lentivirus [71]. Musculoskeletal complications have not yet been reported in Dent-2 patients.

#### Growth failure

Severe post-natal growth retardation is a hallmark of Lowe syndrome and is unrelated to the level of renal insufficiency or bone disease [7, 49]. By 3 years of age, the mean height of Lowe syndrome patients has already fallen to the third percentile, and it continues to fall so during development [13]. Of note, patients with Dent-2 disease also show mild growth retardation, which is not observed in Dent-1 patients, supporting the concept that Dent-2 disease is a mild manifestation of Lowe syndrome [7]. Hou reported improved growth during growth hormone therapy in a Lowe syndrome patient with severe stunting [68]. In view of the extremely delayed puberty (bone age 6 years at the chronological age of 17 years), the impressive treatment response in Hou’s patient may have been due to growth hormone deficiency, which the author did not rule out [68].

#### Oral and dental manifestations

Several case reports have documented dental anomalies in Lowe syndrome patients, including enamel hypoplasia, dysplastic dentin formation, and delayed tooth eruption, the latter being associated with eruption cysts [72]. Some patients develop gingival hyperplasia as a complication of anti-epileptic therapy. Orthodontic complications arise from palatal constriction, crowded teeth, skeletal malocclusion, underdeveloped mandibule, and impacted permanent teeth [73].

#### Hemostasis

Lowe syndrome patients frequently require surgery (e.g., lens extraction, scoliosis correction, dental surgery). This has led to the recognition of a bleeding disorder in Lowe syndrome [74], which is characterized by impaired primary hemostasis when examined in vitro using the PFA-100® platelet function analyzer system (Siemens Healthcare, Erlangen, Germany), while the results of other platelet aggregation tests are normal. These findings may reflect impaired early activation of platelets, i.e., platelet adhesion and shape change, caused by disturbed RhoA-dependent signaling in OCRL-1 deficiency, and have been confirmed in other studies [20]. In addition, mild thrombocytopenia has been noted in around 20 % of patients [20, 74]. Prothrombin time (PT) and activated partial thromboplastin time (aPTT) are normal, as are fibrinogen levels and van Willebrand factor. Tranexamic acid has been observed to ameliorate platelet dysfunction in Lowe syndrome patients (D. Böckenhauer, personal communication).

#### Sexual development

Cryptorchidism is reported in about one-third of Low syndrome patients [32]. Puberty is normal in the majority of patients, as are testosterone levels [13]. Fertility may be reduced due to peritubular fibrosis and azoospermia [75].

#### Dermatological findings

Benign cystic lesions in the skin have been reported in several Lowe syndrome patients. These resemble eruptive vellus hair cysts [76] but may also originate from mature hair follicles [77]. Won et al. reported large epidermal cysts located on the scalp [78]. The etiology of these findings is unclear but has been related to increased extracellular concentrations of lysosomal enzymes [76].

## *OCRL* gene analysis

### Variant spectrum

**Fig. 2:**
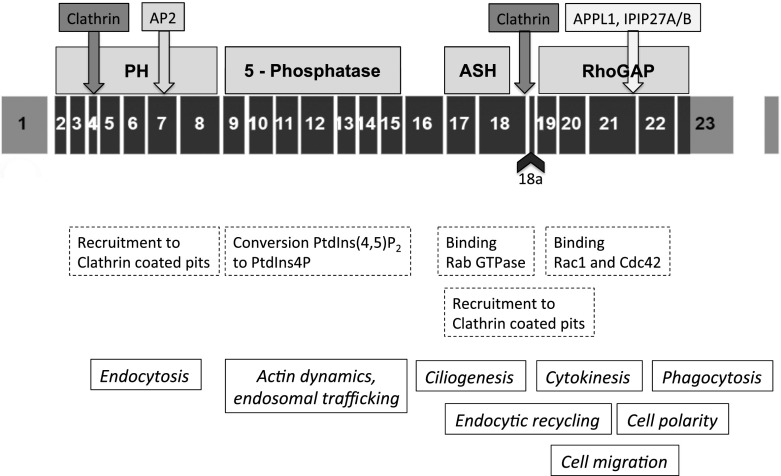
Structure and function of phosphoinositide (4,5)P_2_ 5-phosphatase (OCRL-1). *Black boxes* Exome structure of the *OCRL* gene, including the alternatively spliced exon 18a,* shaded boxes* domains/binding sites of the OCRL-1 protein,* dashed boxes* functions related to the different domains, *text in italics* intracellular processes involving OCRL-1.* PtdIns* Phosphoinositide,* PH* N-terminal pleckstrin homology domain,* 5-phosphatase* 5-phosphatase domain,* ASH* ASPM–SPD2–Hydin domain,* RhoGap* C-terminal (catalytically non-active) Rho GTPase activating-like domain

The *OCRL* gene (Fig. 2) is located on Xq25-26 and comprises 24 exons occupying 52 kb [79]. The coding region includes exons 1–23. Alternative splicing of exon 18a in the brain enlarges the OCRL-1 protein of 893 amino acids by eight (in frame) additional amino acids and improves clathrin binding [79, 80]. *OCRL* nucleotide and amino acid numbering has recently been updated based on the data of Hichri et al. [18].

More than 200 different *OCRL* variants have been described, however in 10–20 % of patients with suspected Lowe syndrome no variant is found [18]. In their study of* OCRL1* gene mutations and clinical and biochemical phenotypes of Lowe syndrome, Hichri et al. reported that 63 % of their Lowe syndrome patients displayed frameshift, nonsense, or splice defects leading to mRNA decay or premature termination of the resultant OCRL-1 protein, while missense variants and gross deletions accounted for 33 and 4 % of the cases, respectively [18]. Of the milder affected Dent-2 patients, 43 % carried frameshift and nonsense variants. Termination variants in Dent-2 disease affect only the first seven exons, whereas they concentrate in exons 8–23 in classic Lowe syndrome [8, 18, 20] (Fig. 2). Other variant types are scattered throughout the *OCRL* gene and unrelated to the Dent-2 disease or Lowe syndrome phenotype. No variant affecting the alternative exon 18a has been reported to date.

Hichri et al. also measured phosphoinositide (PtdIns) (4,5)P_2_ 5-phosphatase (OCRL-1) activity in fibroblasts from patients with Lowe syndrome and Dent-2 disease and found an 80–90 % decrease compared to controls, irrespective of variant type or clinical phenotype [18].

Variants in *OCRL* that are repeatedly observed may either reflect a founder effect or repeat de novo events. As outlined by Recker et al. [20], the fitness of Lowe syndrome patients to reproduce is so low that the half-life of a novel *OCRL* variant is less than two generations, and after four generations more than 90 % of variants will have disappeared. Therefore, the occurrence of an identical variant in unrelated families is most probably due to coincidence, which is in line with the observation that there are no *OCRL* variants predominating in a specific ethnic background.

### Genotype–phenotype correlation

In most cases, the type of variant in Lowe syndrome/Dent-2 disease cannot be correlated to clinical severity. This is illustrated by two missense variants (p.Ile274Thr, p.Arg318Cys) associated with both the mild Dent-2 phenotype and the classic Lowe syndrome phenotype, even within the same family [18].

Recker et al. [20] recently reported a patient with a p.Asp523Asn variant who presented with the cerebral and renal manifestations of Lowe syndrome, while cataract was first noted at the age of 10 years. The same variant was reported by Tosetto et al. [81] in two brothers with the milder Dent-2 phenotype who developed cataract and megalocornea only at the ages of 5 and 8 years, respectively. These observations suggest that the p.Asp523Asn variant exhibits some genotype–phenotype correlation.

## Female carriers

Based on the large study by Hichri et al. [18] about two-thirds of Lowe cases are transmitted by maternal carriers. As in other X-linked diseases, carriers may show a mild phenotype that might aggravate in cases of unfavorable lyonization. In post-pubertal female carriers harboring *OCRL* variants, slitlamp examination invariably reveals punctuate white to gray opacities, distributed in a radial fashion in all layers of the lenticular cortex. This finding can be used for genetic counseling [82].

Manifestation of a more complete phenotype has been reported in a total of ten cases and has been attributed to either cytogenetic abnormalities (reciprocal translocation involving the X-chromosome), a 45,X karyotype, uniparental disomy, or an extremely skewed X-inactivation [83].

## Genetic counseling and prenatal diagnosis

Lowe syndrome/Dent-2 disease can be attributed to a de novo variant in around one-third of all cases. Germline mosaicism for a single-point variant has been reported in five Lowe syndrome families.

In families with a known *OCRL* variant, genetic diagnosis can be performed following chorionic villi or amniotic fluid sampling [84]. Suchy et al. reported prenatal diagnosis by measuring PtdIns(4,5)P_2_ 5-phosphatase activity in cultured amniocytes [85]. However, the lack of genotype–phenotype correlation and the fact that Dent-2 patients and patients with Lowe syndrome have comparable PtdIns(4,5)P_2_ 5-phosphatase activity [18, 86] limit prenatal diagnosis with respect to disease severity. Other parameters that can be used for prenatal screening are elevated maternal serum and amniotic fluid alpha-fetoprotein [87], or the presence of fetal cataract on ultrasonography images [11]. Increased nuchal translucency has recently been reported in two fetuses with Lowe syndrome [88].

## OCRL-1 function

Phosphoinositides play a central role in the regulation of diverse cellular processes, including gene expression, cytokinesis, cell motility, actin cytoskeleton remodeling, membrane trafficking, and cell signaling [33]. Of the seven phosphoinositides identified to date, which differ in the reversible phosphorylation at the 3′, 4′, and 5′ positions of the inositol ring, phosphatidylinositol(4,5)bisphosphate [PtdIns(4,5)P_2_] is the most abundant [33]. Each phosphoinositide has its own unique subcellular distribution, and most organelles appear to be enriched in a specific phosphoinositide. Changing of phosphoinositide species can lead to a switch in compartment identity (e.g., maturation of endosomes) and promote directionality of membrane traffic between distinct compartments [33]. Phosphoinositide kinases and phosphatases play a central role in the regulation of these processes.

OCRL-1 [PtdIns(4,5)P_2_ 5-phosphatase] is one of ten human inositol 5-phosphatases [89] and is expressed in all human cells except cells of hematopoietic origin [90]. OCRL-1 is a multi-domain protein comprising an N-terminal PH (pleckstrin homology) domain, a central 5-phosphatase domain, an ASH (ASPM–SPD2–Hydin) domain, and a C-terminal (catalytically non-active) RhoGAP (Rho GTPase activating)-like domain [89] (Fig. 2).

**Fig. 3:**
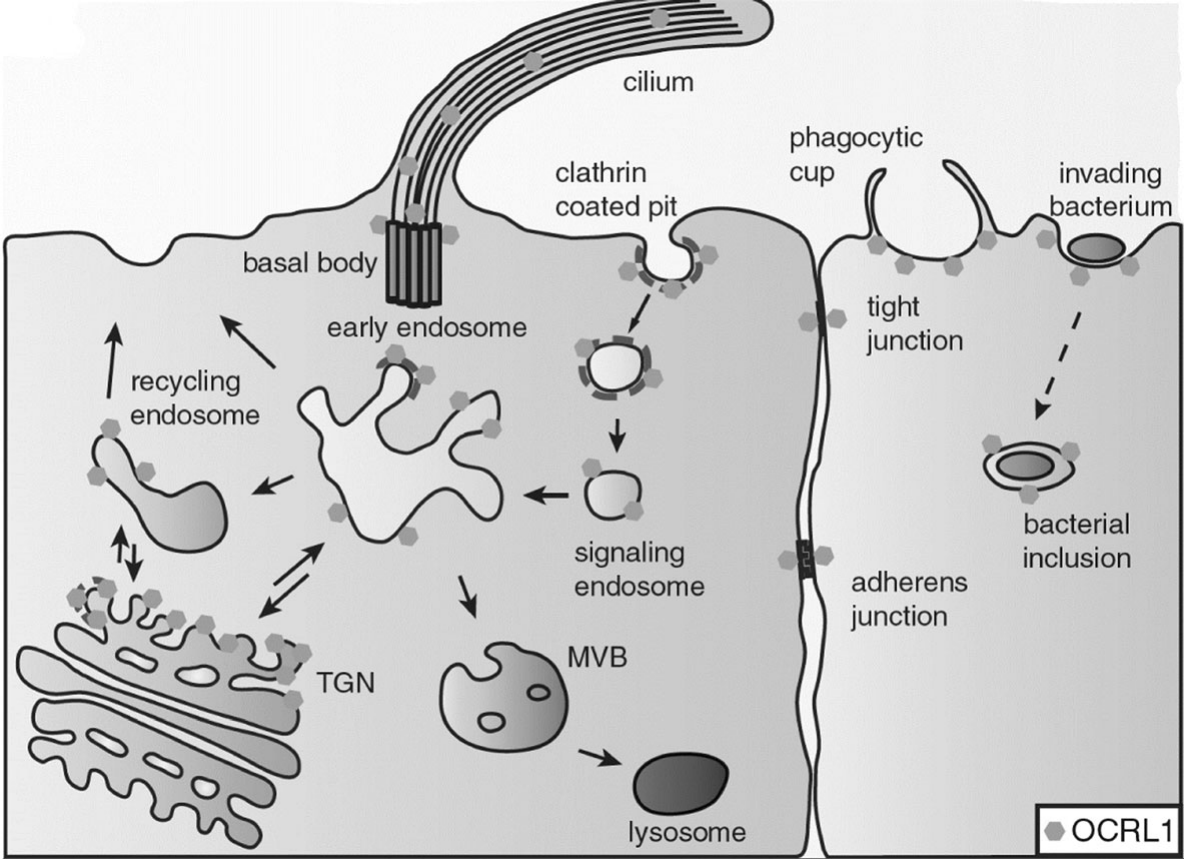
Subcellular localization of OCRL-1. Cartoon summarizing subcellular expression of OCRL-1 (hexagones).* TGN* Trans-Golgi network,* MVB* multivesicular body (modified from Mehta et al. [33], with permission)

In recent years, much progress has been made in terms of understanding the role played by OCRL-1 in many processes of cell metabolism (Fig. 3). These have recently been reviewed in depth by Mehta et al. [33] and Pirruccello et al. [89].

OCRL-1 plays a major role in membrane and endosomal trafficking and is present in clathrin-coated pits on the cell surface, on different types of endosomes and the trans-Golgi network. In clathrin-mediated endocytosis, ORCL-1 is necessary for closure of the newly formed endocytic vesicle [91]. In proximal tubular cells, defective recycling of the megalin receptor after endocytosis [41] accounts for the characteristic shedding of LMW protein in Lowe syndrome and Dent-2 patients (Fig. 1). Defective OCRL-1 leads to increased amounts of PtdIns(4,5)P_2_ on early endosomes, thereby promoting actin accumulation on the endosomal surface [41]. Indeed, decreasing PtdIns(4,5)P_2_ accumulation through the inhibition of PtdIns4P 5-kinases in *OCRL* knock-down cells results in improved endocytosis and diminished F-actin formation [41]. OCRL-1 is also involved in the actin remodeling necessary for phagosome formation at the cell surface [33].

OCRL-1-deficient fibroblasts migrate poorly and show defective cell adhesion [92], again most likely due to dysregulation of the actin cytoskeleton [33]. Cell polarization is also impaired in OCRL-1 deficiency, possibly related to disturbance of adherens and tight junctions [33].

OCRL-1 has been demonstrated in primary cilia [33]. Polarized membrane traffic during cilia formation is regulated via OCRL-1 [93] and fibroblasts from Lowe syndrome patients or cell lines with knocked-down *OCRL* fail to form functional primary cilia [93]. The Rho GTPase binding domain of the OCRL-1 molecule has a critical role in the polarized vesicle processing necessary for cilia formation by interacting with the small GTPase regulators Rab1 and CDC42 [93]. The exclusive expression in the CNS of the extended OCRLb-1 isoform, characterized by increased clathrin binding and the presence of OCRL-1 in neuronal clathrin-coated vesicles from synaptosomal preparations, underscore the importance of clathrin-dependent trafficking for neuronal function [94].

Although at first glance OCRL-1 participates in a large number of cellular processes that are apparently distinct from one another, Mehta et al. have proposed “membrane trafficking” and “actin cytoskeleton remodeling” as the two unifying mechanisms for OCRL-1 action [33].

## Lowe syndrome or Dent-2 disease?

As already noted in this review, there is overlap in clinical signs between patients with Lowe syndrome and those with Dent-2 disease, with the latter possibly presenting with extra-renal features of Lowe syndrome (peripheral cortical lens opacities, stunted growth, mild intellectual impairment, elevation of serum creatine kinase/lactate dehydrogenase, implying that Dent-2 disease represents a mild form of Lowe syndrome [7, 8]. This clinical observation is supported by a recent study on fibroblasts from patients with Lowe syndrome and Dent-2 disease in which Montjean et al. [86] demonstrated an intermediate phenotype of Dent-2 fibroblasts in terms of the F-actin network, alpha-actinin, and primary cilia. Of note, PtdIns(4,5)P_2_ was elevated in cells from patients with Lowe syndrome and Dent-2 disease, and it did not differ between these groups. This result is in line with Hichri et al.’s observation that PtdIns(4,5)P_2_ 5-phosphatase in fibroblasts does not distinguish Dent-2 disease from Lowe syndrome [18].

Furthermore, the description of patients with Lowe syndrome without any ocular involvement [17] and the presentation of two pairs of brothers with discordant clinical phenotypes, one with Lowe syndrome and the other with Dent-2 disease [18], indicates that there are individual differences in the ability to compensate for the loss of enzyme function. It has been suggested that this occurs through INPP5B, an inositol 5-phosphatase which shares nearly all functional domains with OCRL-1 [89]. Indeed, *ocrl* knock-out mice are phenotypically normal as long as murine *inpp5b* is present. As murine *inpp5b* and human *INPP5B* differ in terms of gene transcription and splicing, *inpp5b* may compensate for *ocrl* deficiency in mice, while *INPP5B* does not fulfill this role in man [95]. Indeed, double knock-out of *inpp5b* and *ocrl* resulted in embryonic lethality, while double knock-out with expression of human *INPP5B* in mice using a bacterial artificial chromosome created a phenotype resembling Lowe syndrome/Dent-2 disease (postnatal growth failure, LMW proteinuria, and aminoaciduria) [96]. It is questionable, however, whether this mechanism does explain the phenotypic differences between patients with Dent-2 disease and those with Lowe syndrome as Montjean et al. observed identical expression not only of *OCRL* but also of *INPP5B* at the RNA and protein levels in fibroblasts from both Dent-2 and Lowe syndrome patients [86].

## Conclusions

Using the presence of an *OCRL* variant for case definition, it has become clear that there is a disease spectrum spanning from an isolated tubulopathy (Dent-2 disease) to the most severe presentation of the oculocerebrorenal syndrome described by Lowe et al. in the 1950s. Much progress has been made in terms of understanding the many functions of OCRL-1 in cell metabolism and, consequently, of understanding the multi-organ manifestations of the disease. However, the factors determining disease severity have not yet been clarified. If found they might offer novel therapeutic approaches for this debilitating disease.
